# Editorial: Transforming chronic disease treatment with AI and big data

**DOI:** 10.3389/fmolb.2026.1848975

**Published:** 2026-04-28

**Authors:** Xiaoyan Xing, Zhuolun Sun, Kuo Yang, Shizhang Wei

**Affiliations:** 1 Institute of Medicinal Plant Development, Peking Union Medical College and Chinese Academy of Medical Sciences, Beijing, China; 2 Medizinische Klinik und Poliklinik IV, Klinikum der Universität München, Ludwig-Maximilians-Universität München, Munich, Germany; 3 School of Computer Science & Technology, Beijing Jiaotong University, Beijing, China; 4 Department of Pharmacy, National Cancer Center, National Clinical Research Center for Cancer/Cancer Hospital, Chinese Academy of Medical Sciences and Peking Union Medical College, Beijing, China

**Keywords:** artificial intelligence, big data, biomarker discovery, chronic disease management, personalized medicine

Chronic diseases represent a leading global health burden, characterized by complex etiology, prolonged duration, and significant heterogeneity in clinical presentation and progression. Traditional management strategies, often reliant on population-level evidence and standardized protocols, struggle to address the nuanced, individual-specific dynamics inherent to conditions such as cancer, diabetes, cardiovascular disorders, and metabolic liver diseases. The integration of Artificial Intelligence (AI) and Big Data analytics is heralding a transformative shift, moving healthcare from a reactive, one-size-fits-all model toward proactive, precise, and personalized care. The Research Topic of studies summarized herein exemplifies this paradigm shift, showcasing innovative applications of computational tools across diverse chronic conditions.

A central theme across these works is the leveraging of high-throughput omics data and clinical records to discover novel biomarkers and elucidate disease mechanisms. In liver hepatocellular carcinoma (LIHC), the identification of *DNASE1L3* as a stage-associated biomarker through integrated transcriptomic and clinical data analysis provides a tangible tool for refining prognostic assessment (Xue et al.). Similarly, in clear cell renal cell carcinoma (ccRCC), bioinformatics pipelines pinpointed *CCL5*, *LOX*, *C3*, and *PLG* as key genes linked to tumor progression and patient outcomes (Xie et al.). Beyond oncology, research into type 2 diabetes mellitus (T2DM) uncovered *PPP1CA* and *CTSD* as asparagine-related biomarkers involved in immune-metabolic crosstalk (Xia et al.), while studies in atherosclerosis and atrial fibrillation highlighted shared mitochondrial-immune pathways centered on *MRPS23* (Dai et al.). These discoveries, powered by differential expression analysis, weighted gene co-expression network analysis (WGCNA), and machine learning, underscore the capacity of data-driven approaches to reveal critical molecular drivers often obscured in conventional analyses.

Another pivotal advancement is the development of AI-powered predictive models for diagnosis, stratification, and outcome prediction. The work on non-alcoholic fatty liver disease (NAFLD) demonstrates how incorporating volatile organic compound (VOC) profiles with traditional clinical data significantly enhances detection accuracy using machine learning algorithms (Chen et al.). In the context of T2DM complications, a specialized model (ASBPredictor) was developed to accurately distinguish between asymptomatic bacteriuria and symptomatic urinary tract infections, a clinically relevant challenge with direct implications for antibiotic stewardship (Liu et al.).

The scope of AI applications extends to optimizing therapeutic strategies and understanding treatment mechanisms. In primary aldosteronism, AI models show promise in improving screening, diagnostic specificity, subtype classification, and predicting treatment responses (Xu et al.). Meanwhile, an integrative approach combining network pharmacology, bioinformatics, and *in vivo* validation elucidated the multi-target, anti-inflammatory mechanism of the traditional Chinese medicine Qinghuo Rougan Formula in uveitis, bridging traditional knowledge with modern systems biology (Jing et al.).

Despite the remarkable progress illustrated by these studies, significant challenges remain on the path to clinical integration. Key Research Topic include the need for larger, multi-center datasets to improve model generalizability, the “black-box” nature of some complex AI algorithms requiring enhanced interpretability, and the necessity for rigorous real-world validation in diverse clinical settings. Future research must prioritize the development of robust, transparent, and ethically deployed AI tools that complement clinical expertise.

In conclusion, the convergence of AI and Big Data is fundamentally reshaping the landscape of chronic disease research and management. The works highlighted here—spanning biomarker discovery, predictive modeling, mechanistic elucidation, and therapeutic optimization—collectively chart a course toward a future where healthcare is increasingly predictive, preventive, personalized, and participatory. As computational methodologies continue to evolve and integrate with multimodal health data, their potential to improve patient outcomes, enhance clinical decision-making, and reduce the overall burden of chronic diseases will only expand. The journey from population-based paradigms to truly personalized medicine is well underway, driven by the innovative fusion of clinical insight and technological prowess.

